# Preventive and Tumor-Suppressive Effects of Lactobacillus Paracasei X12 in Rat Model of Colorectal Cancer

**DOI:** 10.22037/ijpr.2019.112135.13547

**Published:** 2020

**Authors:** Sayyid Ali Mousavi Jam, Mohammad Morshedi, Ahmad Yari Khosroushahi, Amir Taher Eftekharsadat, Maedeh Alipour, Beitullah Alipour

**Affiliations:** a *Drug Applied Research Center, Tabriz University of Medical Sciences, Tabriz, Iran.*; b *Faculty of Medicine, Tabriz University of Medical Sciences, Tabriz, Iran. *; c *Department of Medical Nanotechnology, Faculty of Advanced Medical Sciences, Tabriz University of Medical Sciences, Tabriz, Iran. *; d *Department of Pathology, Imam Reza Hospital, Tabriz University of Medical Sciences, Tabriz, Iran. *; e *Department of Community Nutrition, Faculty of Nutrition and Food Sciences, Tabriz University of Medical Sciences, Tabriz, Iran.*

**Keywords:** Apoptosis, Colorectal cancer, Lactobacillus, Probiotics, Tumor-suppressive, Cell proliferation

## Abstract

The paper in hand seeks to evaluate the tumor-suppressive and apoptotic effects of *L. paracasei* X12 in 1, 2-dimethylhydrazine (DMH)-induced rat colon carcinogenesis. The rats were divided into three groups (n = 8-12 per group*); L. paracasei* X12 was administrated to these animals for forty weeks. The findings of this study indicated that *L. paracasei* X12 administration prevented severe weight loss in DMH-treated rats. It was also determined that *L. paracasei* X12 administration could prevent the neoplasia incidence, cell proliferation and it also could suppress the tumors’ growth. Additionally, a significant improvement was observed in apoptosis indexes and cell proliferation in probiotic-treated rats. In conclusion, this study provides insights into the therapeutic potential of *L. paracasei* X12 with emphasis on the issue that modulation of apoptosis pathway could leave beneficial effects in the prevention and suppression of colorectal cancer (CRC). However, further studies are in support to clarify the mechanisms involved in the tumor-suppressive effect of probiotics.

## Introduction

Nowadays, being one of the most notable causes of mortality in the world, cancer is considered to be a major health challenge worldwide. Colorectal cancer (CRC) is one of the fatal and most common cancers whose mortality rate varies widely and is expected to be doubled by 2030 (1). Prevention is of high significance for global health strategies as CRC holds a poor prognosis. Hence, preventive intervention research seems to have warranted global health strategies in order to decrease the severity of the incidence and development of CRC (1, 2).

It was demonstrated in the past decade that gut microbiota can bring health benefits to the host (3). Recent evidence has illustrated that gut dysbiosis (imbalanced gut microbiota composition) is closely associated with a number of disorders, including inflammation; epithelial cells dysfunction, and immune system impairment (3-5). Several studies evinced that gut microbiota and their metabolites could affect the initiation and progression of CRC (6-8). The gut microbiota is affected by a variety of factors, especially diet. Probiotics such as Lactobacilli species (*i.e.* live microorganisms that are useful for the body when applied in proper amounts) have various beneficial effects on the host (5, 9-13). 

Recently, the use of probiotics is increasing for the prevention and treatment of diseases (11, 14 and 15). Both *in-vitro* and *in-vivo* researches have divulged the tumor-suppressive effects of probiotics (16-20). Today, the beneficial roles of probiotics in improving CRC have been proposed (16, 21). Notwithstanding the foregoing, mechanisms of probiotics species on CRC are still unclear (17). In addition, there still remains the controversy regarding the effectiveness of probiotics on CRC (16, 22). Therefore, it is possible for the probiotics to play a major role in preventing and suppressing CRC via alleviation of gut pathogens, proliferation and differentiation of epithelial cells, reducing inflammation (23, 24), and improving antioxidant defense system as well as stimulating mucosal immunity (9, 11 and 25-27). Thus, probiotics may offer therapeutic benefits for CRC treatment.

The main characteristics of cancer are the uncontrolled growth and proliferation of cells and resistance to apoptosis. Apoptosis is a precise cellular program for controlling the number of cells mediated by caspases (28, 29). It is regulated via two (external and internal) pathways. Caspases-2, 8, and 9 are closely connected to pro-apoptotic signals (29). Evidence demonstrated that probiotics consumption may have a beneficial effect on apoptosis mechanism (17, 28). Two studies reported that administration of propionibacteria or L.casei have apoptotic effects on CRC (17, 18). Moreover, another work reported that *Lactobacillus* acidophilus intake could inhibit the expression of the pro-apoptotic protein (Bax) and increase anti-apoptotic protein (Bcl-2) levels (30).

*L. paracasei* is a common probiotic, found abundantly in dairy products. Recently, several studies signaled that *L. paracasei* X12 have had beneficial effects, such as improving dysbiosis as well as inflammatory and immune responses in the host (31-33). Studies demonstrated tumor-suppressive effects of several *Lactobacillus* species such as *L. paracasei* X12 (17, 34-36). Based on these reports, the present study aims to identify the preventive effects of *L. paracasei* X12 compared with CRC and evaluate the effects of *L. paracasei* X12 on the apoptosis pathway and cell proliferation via argyrophilic nucleolar organiser region-associated proteins (AgNORs) in 1, 2-dimethylhydrazine (DMH)-induced colorectal carcinogenesis in rats with CRC.

## Experimental

*Animals*

Thirty-six male Wistar rats (160±20 g, 6 weeks old) were obtained from the Pasteur Institute of Iran. The rats were housed in cages of four in a controlled condition (temperature: 22–25 °C, humidity: 50–60% and 12 h: 12 h repeating cycle). They had free access to food and water. The care and treatment of all animals were in accordance with the Principles of Laboratory Animal Care (NIH Publication 1986). The present experiment was approved by the Animal Experimentation Ethics Committee of Tabriz University of Medical Sciences, Tabriz, Iran.

*Study design*

The animals were randomly divided into three experimental groups. Each group comprised of 12 rats, as follows: 

Group I (n = 12), Healthy control (HC), which continued to receive single weekly doses of 1 mM EDTA saline until the end of the study.

 Group II (n = 8), DMH treated rats’ control (DC), which received a single dose of DMH (Sigma, St. Louis, MO, USA) subcutaneously (SC) and sterile normal saline intragastrically until the end of the study.

 Group III (n = 12), probiotic treated rats (DP), in which the rats were injected with DMH*. L. paracasei* X12 was being fed to the rats daily for 40 weeks. Body weight (BW) was measured every 8 weeks. Eventually, the rats were anesthetized with sodium pentobarbital (65 mg/kg BW, IP) and were sacrificed by cervical dislocation at the endpoint. After laparotomy, the colon and rectum tissues were removed and cut longitudinally. Then, it was thoroughly washed with cold saline.

*Preparation of probiotic*

The studied probiotic, *L. paracasei* X12 was previously isolated from traditionally fermented dairy (20). In the present work, *L. paracasei* X12 was obtained from TBZMED Biotechnology Research Center (Tabriz, Iran). Probiotic suspension was prepared based on Morshedi *et al.* study (9). Briefly, *L. paracasei* X12 was inoculated in MRS (Man-Rogosa-Sharpe) broth and was maintained in an incubator (48 h at 37 °C). It was isolated from MRS broth by centrifugation (3000 rmp, 4min, and 4 °C); then, it was washed with sterile phosphate-buffered saline (PBS). Fresh bacterial suspensions were suspended in PBS at a concentration of 2 × 10^9^ colony-forming units (CFU)/rat/day and administrated intragastrically every day for each rat in DP group.

*Induction of colon carcinogenesis*

DMH was freshly prepared in 1 mM EDTA with pH adjusted to 7.0 by 1 mM NaOH. Single doses of DMH (30 mg/kg BW, twice a week) were given to SC for 12 consecutive weeks.

*Tumor* *evaluation*

After killing the rats, their colon from the cecum to the anus were harvested and washed with cold saline. The tissues were opened longitudinally and inner surfaces were examined for the presence of the macroscopic lesion. The tumors count, volume, and multiplicity were counted and measured, using Vernier caliper (0.1-mm graduation). 

*Histological examination*

For the histological investigation, tissues and gross lesions were fixed in buffered formalin (10%) and were processed and embedded in paraffin blocks for the histopathological examinations at light microscopy. The grade of differentiation occupying the largest area in the totality of the tumor was assessed according to the World Health Organization (WHO) classification for tumors of the colon and rectum. The assessments were randomized and single-blinded. Histological sections were stained with hematoxylin and eosin (H&E) (37). 

*Evaluation of apoptosis markers by Real-time PCR analysis*

After tumor tissues homogenization, total RNAs of tumor cells were isolated from each rat, using RNX-plus solution (SinaClon, Tehran, Iran) and were used for complementary DNA (cDNA) by PrimeScript RT Reagent Kit (Takara Bio Inc., Tokyo, Japan). cDNAs were used as templates using specific primers. The probes are listed in )Table 1(. All amplification reactions were performed on ABI-step I plus (Applied Biosystems, CA, USA) instrument (triplicate for each sample). 

*Evaluation of Annexin V staining*

For this purpose, tissues with tumor were cut into small pieces and smashed with disposable pestles. The mashed tissues were homogenized by Dispase II (Roche Diagnostics GmbH), using the enzymatic method. Apoptotic cells were identified by staining with Annexin-V and propidium iodide (PI), using apoptosis detection kit (BioLegend Inc., San Diego, USA). Finally, 400 μL of Annexin V Binding Buffer was added to each tube and analyzed by flow cytometry, used to examine the expression of large numbers of specific markers and indicators of apoptosis. Quadrant settings were fixed with untreated, single-stained controls and copied to dot plots of the treated cells. Data analysis was conducted using CELL Quest Pro software (BD Biosciences, San Jose, CA, USA). 

*Detection of apoptosis with M30 CytoDEATH cells*

Apoptosis was identified by M30 CytoDEATH antibody Kit (Peviva AB, Bromma, Sweden), according to the manufacturer’s instructions. Evaluation of M30 staining was performed by two independent researchers. M30 positivity was identified as brown cytoplasmic staining per five fields at a magnification of 100x under a light microscope (Olympus BX64, Optical.Co. Ltd, Japan). At least, 1000 epithelial cells were counted in each group of slides and the M30 positive cells were represented as a percentage of the total number of counted cells and disclosed the apoptotic index. 

*Evaluation of cell proliferation by AgNOR staining*

AgNORs were identified by silver nitrate staining. AgNORs staining was performed, using the method of Murray *et al*. (1989). Briefly, AgNOR Stained slides were observed under the light microscope (Olympus BX64, Optical.Co.Ltd, Japan) at a magnification of 40x by two investigators with no knowledge on the tumor type, grade, and group’s properties. AgNOR in one hundred cells were counted and the mean number of dots per nuclei was considered separately for each group. The count was the percentage of nuclei exhibiting five or more AgNOR granules/nucleus/100 cells and represented proliferative activity. Tumors with proliferative activity count were characterized as percentages of AgNOR (pAgNOR). High proliferative activity was determined by PAgNOR ≥ 8%.

*Statistical analysis*

Results were obtained from 8-12 rats in each group. All data were analyzed using SPSS (Version 23). One-way analysis of variance (ANOVA) followed by *post-hoc* Tukey’s test was used for comparison of significance between the groups. Data were considered noticeable at *P *< 0.05.

## Results

*The Effects of L. paracasei X12 on weight gain*

**Figure 1:**
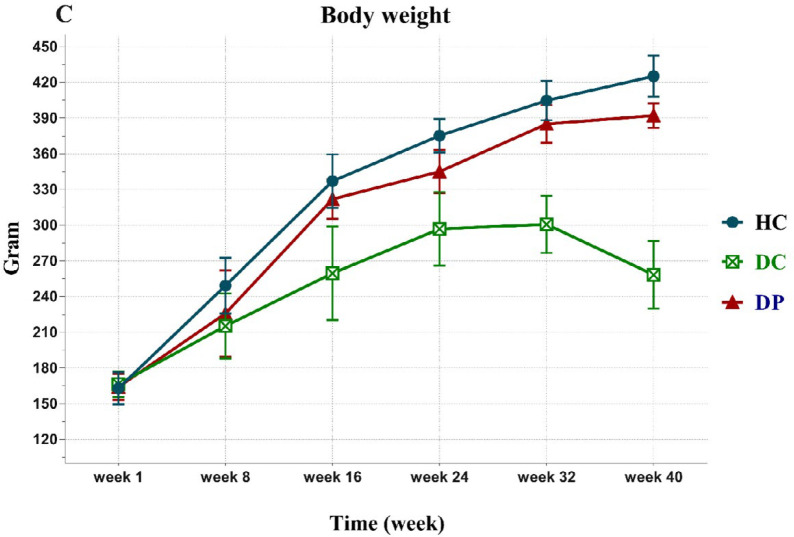
***Effects of the*** **L. paracasei** ***X12 treatment on weight gain after 40 weeks***

The BW of the rats did not differ notably among the three groups at the beginning of the intervention (Figure 1). At the end of the 8^th^, 16t^h^, 32^th^, and 40^th^ weeks, the BW of the DC group strikingly decreased, compared to the HC group. Probiotic intake had an important effect in preventing weight loss in these weeks (*P *< 0.001). The greatest weight loss was observed between the 32^th^ to 40^th^ weeks after DMH injection group (DC). Weight loss in the treated group (DP) was prevented (*P* < 0.001). In addition, a remarkable increased BW was progressively observed in the HC and DP groups.

*The Effects of L. paracasei X12 on physical changes Tumors*

In this work, the incidence of colon tumor was 100%, following a 12-week DMH injection to the rats. In the DC group, the majority of tumors were adenocarcinoma (Table 2). In the Probiotic-treated group (DP), only 4/12 of rats had lesion with moderate dysplasia and tubular adenoma. Besides, the tumor incidence was prominently decreased to ~66% in the DP group (Table 2). 

**Figure 2:**
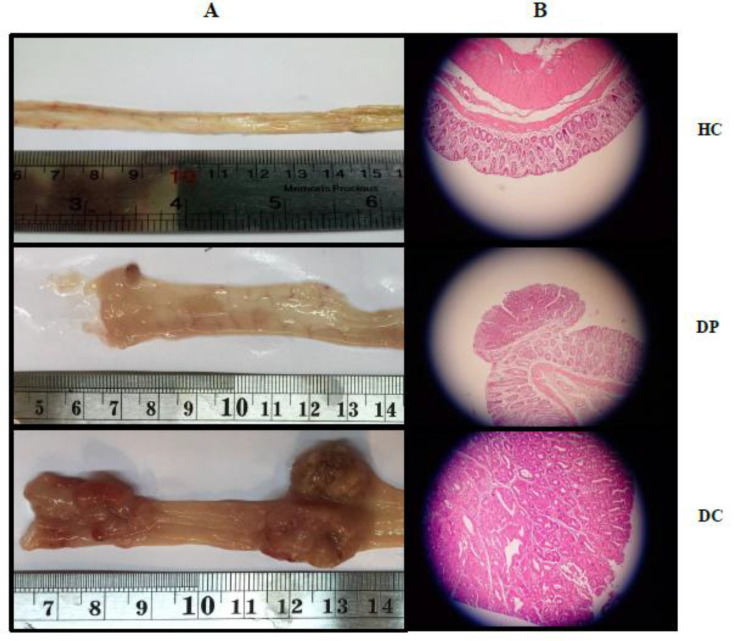
Effects of *L. paracasei* X12 on macroscopic appearance of tumor incidence, multiplicity and volume in colon of rats (A). Representative photomicrographs of histological (hematoxylin and eosin) cross-sections of colons tissue with magnification 40X from the treated and untreated rats (B). Normal colorectal tissues (HC group), colorectal adenoma (DP group) and colorectal adenocarcinoma (DC group). HC: healthy control group; DC: DMH alone group; DP: DMH induced rats treated by the L.paracasei X12

Additionally, injection of DMH in the rats led to a significant increase in the size, multiplicity, and volume of the tumors, compared to the DP group (*P* < 0.001). Probiotic administration in the rats with CRC reduced both volume and number of the tumors (*P* < 0.001). As shown, supplementation with the probiotic could inhibit tumor growth (Figure 2A).

*The Effects of L. paracasei X12 use on Histopathological changes in the tumor*

Histopathological analysis demonstrated that the colon of animals in the HC group had normal histoarchitecture. In the DC group, the signs of dysplasia (adenoma and adenocarcinoma) were observed, while in the DP group, the histopathological analysis revealed just a few adenomas (Table 2). However, there were many adenocarcinomas in the colon of DMH-induced rats. Probiotics administration could prevent such increases in cell proliferation (Figure 2B).

*The Effects of L. paracasei X12 use on cell proliferation*

AgNORs was measured to understand the cell proliferation protective effects of *L. paracasei* X12 over DMH-induced colorectal carcinogenesis in the nuclei of all the colon tissues of the rats (Figure 4B). 

There was an eminent statistical difference between the DC and HC groups, regarding mean AgNORs concentration (Table 3). Furthermore, in comparison with the DC group, probiotic consumption (DP group) resulted in a decreased level of mean AgNORs (mAgNORs) and reduced level of AgNOR (pAgNOR) (*P *< 0.05). As stated, the supplementation with probiotic could prevent the increase in cell proliferation (Table 3)

*The Effects of L. paracasei X12 use on apoptosis markers*

*Expression of apoptosis markers*

**Figure 3:**
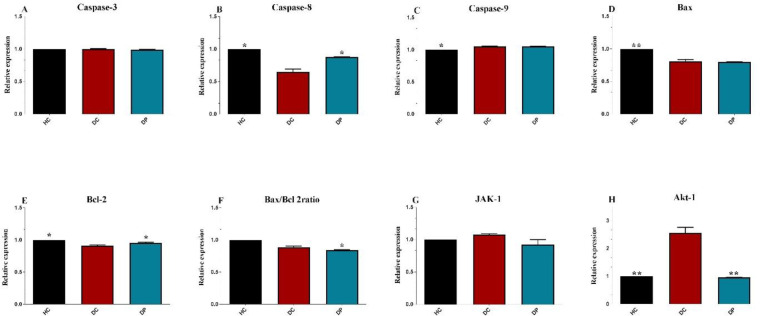
Effects of the L. paracasei X12 treatment on apoptosis markers expression. HC: healthy control group; DC: DMH alone group; DP: DMH rats treated by the L.paracasei X12

The effects of *L. paracasei* X12 administration on the pro - and anti-apoptotic proteins were investigated (Figure 3). Following DMH injection, the levels of caspase-9, Bcl-2 and Bax/Bcl-2 ratio greatly decreased; however, caspase-8, caspase-3, Bax and Janus kinase 1 (Jak-1(, and Akt-1 levels obviously increased compared to HC group. Meanwhile, there was a major decrease in caspase-3, caspase-9, and Bax expression; however, this was fundamentally increased in caspase-8, Jak-1, Akt-1, Bcl-2 and Bax/Bcl-2 ratio, compared to DP group (*P *< 0.005). 

*Annexin V and PI staining*

**Figure 4:**
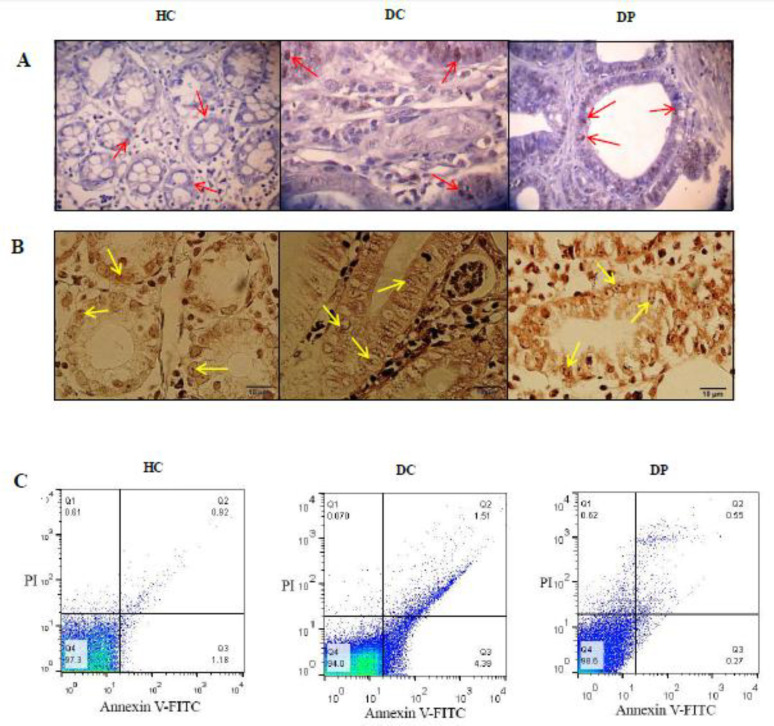
***The colonic histology scores (M30 CytoDEATH cells, AgNOR staining and Annexin V staining) of different groups of rats were assessed (n=8-12 per group). Typical M30 CytoDEATH stains of apoptotic cells. Red arrows indicate to positive-stained cells present in this field. Magnification scale is 1000X (A). The AgNORs are situated within the nucleoli (oil immersion, x 1000). Note numerous black dots (yellow arrows) scattered throughout the nucleus which can be easily discriminated as well as complete lack of staining artefacts (AgNOR, bar = 10 µm) (B). The single-cell suspension was prepared and apoptosis was detected by annexin-V fluorescein isothiocyanate through flow cytometry. (C). The Q1 quadrant represents unviable cells. The Q2 quadrant represents cell that are in late apoptosis. The Q3 quadrant represents cells in early apoptosis. The Q4 quadrant represents viable cells. HC: healthy control group; DC: DMH alone group; DP: DMH rats treated by the L.paracasei X12***

The relative apoptosis promoting activities of probiotic *L. paracasei* X12 was assessed by Annexin- V and PI staining, followed by flow cytometry. There was a crucial difference in the proportion of Annexin-V positive between the groups. Quantification of annexin V and PI positive cells reflected a critical difference in late (Q2) and early (Q3) cell apoptosis or necrosis between DC and DP groups (Figure 4C). 

*M30 CytoDEATH cells*

Following induced CRC via DMH, M30 CytoDEATH cells number increased significantly compared to the HC group (Tab. 3). Additionally, in comparison with the DC group, probiotic therapy could relevantly increase M30 CytoDEATH cells and apoptotic index (Table 3) in the colon tissue after 40 weeks of treatment (Figure 4A). 

## Discussion

There is a growing evidence that the consumption of probiotics may have therapeutic and protective effects on CRC (16-19, 21, 35). Therefore, the present work was conducted to explore the preventive effect of *L. paracasei* X12 opposing the progress of CRC in the rats. It was signified that the use of *L. paracasei* X12 can prevent severe weight loss in DMH-induced rats. The obtained results also elucidated that the administration of L. *paracasei* X12 can prevent the onset or the growth (cell proliferation) of the tumors. It was also observed that probiotic intake could decrease the size, multiplicity, and volume of the tumors. *L. paracasei* X12 use resulted in the increased apoptotic markers in the colon. 

The administration of *L. paracasei* X12 can prevent severe weight loss induced by DMH. In the DM group, the increase in BW was not similar to the other groups. Li *et al*. (38) have also reported that BW of the rats with DMH injection was considerably reduced, compared to the healthy rats. One of the most critical reasons for weight loss after induction of cancer is the loss of muscle mass (39). In several studies on CRC, it was found that inadequate muscle mass is a strong determinant of mortality (40). On the other hand, low muscle mass could lead to mitochondrial dysfunction (41), which may accelerate cancer progression (42). BW loss could be the result of degeneration of lipids and proteins and changes in the metabolism of the body to provide energy for hyperplastic cells (43). As denoted previously (43, 44), it is also believed that the administration of probiotics could improve weight loss induced by cancer. 

To the best of knowledge, it was illustrated that *L. paracasei* X12 use could prevent the onset of the tumors caused by DMH injection in most of the rats. In some cases, it was observed that *L. paracasei* X12 use has suppressive effects on the incidence, size, multiplicity, and volume of the tumors. Furthermore, tumors were adenocarcinomatous in the majority of the DMH-induced rats, while in the treated rats (DP) just four rats had adenoma. Other data also illustrated that the probiotic intake resulted in reduced mAgNORs and pAgNORs levels of the colon cell proliferation in comparison with DMH-induced rats. In this regard, Grimoud *et al*. (45) specified that the reason for the decrease in cell proliferation after probiotics (B.breve R0070 and L.lactis R1058) administration *in-vitro* is the increase in cellular differentiation and reduction of the inflammation. In another study, Chen *et al*. (15) reported that administration of L. acidophilus NCFM (10^8^ CFU/mice) in female BALB/cByJ mice for 28 days led to 35.5% decrease in mean tumor volume as well as the increase in the number of apoptotic cells in treated vs. untreated mice. Zhang *et al. *(35) also implied that L. salivarius (5 × 10^10^ CFU/kg for 40 weeks) substantially reduced the size, multiplicity, and volume of the tumor. Meanwhile, L. salivarius could modulate the gut microbiota of the DMH-induced rats. The proposed mechanism in that study was the increase in the activity of the immune system via improving the gut microbial composition. In another study, Konishi *et al*. (17) revealed that probiotic-derived ferrichrome administration prevented the cell growth of colon cancer cells, while it did not have any effect on cell growth of non-cancerous cells of the small intestine in the rats. Notably, the tumor-suppressive effect of ferrichrome was stronger than anticancer drugs. In contrast to this study, Arthur *et al*. (22) suggested that VSL#3 probiotic intake (10^9^ CFU/animal/day) for 17 weeks could not decrees inflammation and tumorigenesis in a mouse model of colitis-associated CRC (induced by azoxymethane injection). In addition, there was no notable difference in the tumor penetrance and dysplasia score among the experimental groups. However, tumor multiplicity was meaningfully decreased in VSL#3-treated animals. According to the evidence, among the proposed mechanisms for CRC, various probiotics may reduce inflammation and strengthen immune responses (46-48) by improving the gut microbial composition and maintaining the integrity of the intestine (49-51) which is probably the main reason for inhibition of the cell proliferation and prevention or suppression of colon tumors (52, 53).

The present study marked that *L. paracasei* X12 use can ameliorate the expression of apoptosis index in the colon cells. The apoptosis mechanism is one of the most noteworthy pathways against the growth of tumors. This pathway is disrupted in cancerous cells (54). This paper’s finding well demonstrated that *L. paracasei* X12 therapy led to notable improvement in M30 CytoDEATH cells (positive cells). Although recent evidence in different *in-vitro*, *in-vivo* and human clinical trials have implied that the consumption of probiotics can alleviate disease symptoms, especially gastrointestinal disorders and relieve negative symptoms of CRC; however, the exact mechanisms for anticancer and cancer preventive activity of probiotics remain yet unknown (55). One of the possible mechanisms that researchers have proposed for the anticancer effect of probiotics is the induction of apoptosis in cancerous cells via down/up-regulation of key genes in apoptosis and proliferation pathways (56-58) The intrinsic apoptosis pathway was regulated by two main groups of the Bcl-2 proteins, 1) the pro-apoptotic proteins (*e.g*. Bax, Bak, Bad, Bcl-Xs, Bid, Bik, Bim, and Hrk) that promote release of cytochrome c and 2) the anti-apoptotic proteins (*e.g*. Bcl-2, Bcl-XL, Bcl-W, Bfl-1, and Mcl-1) that block the mitochondrial release of cytochrome-c (59). Different studies reflected that some probiotic strains, such as L. bulgaricus, L. rhamnosus GG and B. latis Bb12 can modulate some anti-apoptotic and pro-apoptotic gene expression like Bax and Bcl-2 can stimulate apoptotic protein like caspase-3 can suppress some survival signaling and can produce some metabolites, such as surfactin and short-chain fatty acids which induce apoptosis in colorectal carcinoma cells (60). On the other hand, some investigations detailed that Jak/signal transducer and activator of transcription (STAT) signaling pathway play an outstanding role in the progression of CRC. This pathway possesses such a notable role in several physiological pathways, including cell growth/differentiation, hematopoiesis, immunity, cell survival, invasion, angiogenesis and migration by regulation of some gene expressions, such as Bcl-2, p16ink4a, p21waf1/cip1, and p27kip1, E-cadherin, VEGF, and MMPs (61). Some studies revealed that Akt1, Jak2, and STAT3 signaling pathway can be good potential therapeutic targets in the human CRC treatment due to their activity in numerous parts of tumorigenesis and progression. As well, Ma *et al*. cited that there are particular correlations between STAT3, survivin and Bcl-xl expression levels and also the level of activated phospho-STAT3 (pSTAT3) has been increased in CRC patients (62). However, the growth of cancer cells can suppress through inhibition of Jak/STAT signaling pathway and corresponding apoptosis induction (63).

In this study, treatment with L. paracasei X12 remarkably downregulated anti-apoptotic and proliferation inducer genes like Bcl-2, Jak-1, and Akt-1 and upregulated pro-apoptotic genes like Bax, Cas-3, -9 in DP group in comparison with DC group. Also, administration of L.paracasei prevented from induction and progression of CRC tumors in rats through suppress tumor cell proliferation and induction of apoptosis by triggering both intrinsic and extrinsic apoptosis pathways.

However, the histopathological evaluation of the colon cells illustrated that *L. paracasei* X12 increase apoptotic index and also there is a crucial deference in the percentage of early and late cell apoptosis in the colon tissue, compared with the DMH-alone-exposed rats. There were notable effects in either preventing the onset of tumor cells or suppressing their growth that was probably due to the beneficial effects of the probiotic on various apoptosis pathways and decrease of the cell proliferation. In a similar study, Konishi *et al*. (17) implied that L. casei ATCC334 induces apoptosis (*i.e.* caspase-3) in colon cancer cells *in-vitro* through the activation of c-jun N-terminal kinase (JNK). In addition, TUNEL staining indicated that the number of apoptotic cells in the probiotic-treated cells was higher than the control cells. However, it was mentioned that their results were not investigated *in-vivo*. Another study reported that the administration of L. casei ATCC393 (10^9^ CFU/day) for 13 days significantly inhibited the growth of colon carcinoma cells and diminution in the tumor volume of treated mice (64). They also signaled that L. casei ATCC393 did not substantiate perceptible differences either in CT26 or HT29 cells *in-vitro* using flow cytometry, compared to the control group. Apoptosis of colon cancer cells by Annexin V and PI was reported in another study (64). According to the evidence mentioned above, it can be concluded that apoptotic effects (evaluate different pathways) of probiotics still need to be investigated. However, the final results have been manifested to be desirable in increasing tumor cell death via apoptosis. In another study by Sharma *et al.* (30), intake of L. acidophilus (10^8^ CFU/day) could inhibit the expression of the pro-apoptotic protein (Bax) and increase the anti-apoptotic protein (Bcl-2) levels via reduced oxidative stress (17). Inhibition of the cell proliferation could be due to the increased apoptosis or not as well; since a study reported that reduction of cell proliferation was not through modulating apoptosis. It was demonstrated that the anti-proliferative effect of probiotic was through the decrement of ErbB2 and ErbB3 receptors which resulted in downstream signaling molecules E2F and cyclin D1 (65).

Generally, the used data examplified that *L. paracasei* X12 administration prevented severe weight loss in DMH-induced rats. It was also well demonstrated that *L. paracasei* X12 use resulted in preventing the incidence of the neoplastic cell and suppression of the development of the tumor. Distinguishable changes were observed in the apoptosis markers, flow cytometry index, and cell proliferation in L.paracasei-treated rats, as well. Despite the favorable results obtained in the present study, there were some limitations, including the lack of examination of gut microbial changes that contribute to the clarity of the mechanisms of the tumor-suppressive actions of probiotics administration.

**Table 1:** Primers sequences for RT-PCR amplification

Target genes	Sequence (5′ ➔ 3′)	Amplicon size (bp)	Annealing temperature (°C)
*GAPDH*	F: 5'- CGTGTTCCTACCCCCAATGTATC-3' R: 5'- TAGCCCAGGATGCCCTTTAG-3'	128	57.9
*Bcl*-2	F: 5'- TGGCGATGAACTGGACAACA-3' R: 5'- CCAGTTGAAGTTGCCGTCTG-3'	124	58.6
*Bax*	F: 5'- AGCTGCAGAGGATGATTGCT-3' R: 5'- AGCAAAGTAGAAAAGGGCAACC-3'	128	58.4
*JAK-1*	F: 5'- CTAATCGGACAACCTTTCAGAACC-3' R: 5'- AAATGGCTTGGGAGAGAAGGA-3'	110	58.3
*Akt-1*	F: 5'- AGGCATCCCTTCCTTACAGC-3' R: 5'- CCTCTGAAAACACGCGCTC-3'	127	58.5
*Caspase-3*	F: 5'- TGGAACTGACGATGATATGGCA-3' R: 5'- CTGGATGAACCATGACCCGT-3'	124	58.7
*Caspase-8*	F: 5'- TTTCCGGGTCAACAGGAGCTTG-3' R: 5'- TTGATGGTCACCTCATCCAAAAC-3'	126	60.1
*Caspase-9*	F: 5'- ACATCGAGACCTTGGATGGTG-3' R: 5'- AGTTAAAACAGCCAGGAATCTGC-3'	129	58.6

TM, melting temperature of primers; F, forward primer; R, reverse primer.

**Table 2:** Effect of L. paracasei X12 intake on progression of colon tumors in groups after 40 weeks

Groups	HC n (12)	DC n (8)	DP n (12)
Tumor incidence	^*^ 0/12	8/8	^*^ 4/12
Adenoma (%) (mean)	^*^ 0	6.8%	100%
Adenocarcinoma (%) (mean)	^*^ 0	93.1%	0
Carcinoma (%) (mean)	0	0	0
Tumor volume (mm2) (mean)	^*^ 0	66.6	^*^ 3.4
Tumor multiplicity (mean)	^*^ 0	10.0	^*^ 0.8

Compared to the DC group, *P* < 0.05 was regarded as statistically significant. HC: healthy control group; DC: DMH alone group; DP: DMH rats treated by the L.paracasei X12.

**Table 3:** M30-positive cells and apoptosis index in different cancerous and comparison of mean number of AgNOR (mAgNOR) and proliferative index (pAgNOR) in 100 tumors nuclei in treated/untreated and normal groups

Groups	HC n (12)	DC n (8)	DP n (12)
M30-positive cells (mean ± SD)	^* ^91.41± 9.99	118.3 ± 14.43	^* ^174.1 ± 76.01
Apoptotic index (mean ± SD)	^*^ 0.09 ± 0.01	0.11 ± 0.01	^* ^0.17 ± 0.01
mAgNOR (mean ± SD)	^*^ 1.21 ± 0.07	4.4 ± 0.09	^* ^1.6 ± 0.68
PAgNOR (%)	^*^ 2%	67%	^*^7%

Compared to the DC group, *P* < 0.05 was regarded as statistically significant. . HC: healthy control group; DC: DMH alone group; DP: DMH rats treated by the L.paracasei X12.

## Conclusion

It was elucidated that *L. paracasei* X12 performed anti-proliferative and tumor-suppressive effects in reducing the incidence of CRC. In this regard, the findings of this study suggest that *L*. *paracasei* X12 use prevented severe weight loss and resulted in decreased size, multiplicity, and volume of the adenomas. It was revealed that probiotic intake could ameliorate apoptosis markers in colon cells to a remarkably high level. Probiotic administration seems to be a promising avenue of research in the prevention of CRC. Further investigations are warranted to clarify active bacterial components, and more signaling pathways. Probiotics administration may hold a prominent potential in matters of control and management of CRC. Finally, more researches need to be conducted regarding cancers for the association of nutritional strategies. 
